# Coherent perfect absorption in one-sided reflectionless media

**DOI:** 10.1038/srep35356

**Published:** 2016-10-19

**Authors:** Jin-Hui Wu, M. Artoni, G. C. La Rocca

**Affiliations:** 1 Center for Quantum Sciences, Northeast Normal University, Changchun 130117, China; 2 Department of Engineering and Information Technology & Istituto Nazionale Ottica (INO-CNR), Brescia University, 25133 Brescia, Italy; 3 Scuola Normale Superiore and CNISM, 56126 Pisa, Italy

## Abstract

In optical experiments one-sided reflectionless (*ORL*) and coherent perfect absorption (*CPA*) are unusual scattering properties yet fascinating for their fundamental aspects and for their practical interest. Although these two concepts have so far remained separated from each other, we prove that the two phenomena are indeed strictly connected. We show that a *CPA*–*ORL* connection exists between pairs of points lying along *lines* close to each other in the 3D space-parameters of a realistic lossy atomic photonic crystal. The connection is expected to be a generic feature of wave scattering in non-Hermitian optical media encompassing, as a particular case, wave scattering in parity-time (*PT*) symmetric media.

Scattering from complex potentials and the associated non-Hermitian Hamiltonians^1^ are usually introduced to describe dissipation or decay processes in open systems. Likewise light wave propagation phenomena through media with *complex susceptibilities* are genuine realizations of scattering from localized non-Hermitian potentials and provide a clear illustration of how Hermitian and non-Hermitian processes differ from one another. The optical scattering matrix *S* fully governs the propagation of light and, in particular, one-sided reflectionless (*ORL*) scattering of light waves impinging from “one” direction^2^,^3^,^4^,^5^,^6^ can be associated with a non-Hermitian degeneracy^7^ of the scattering matrix (also known as an exceptional point^8^). More intriguing phenomena appear, however, when coherent waves impinge on “both” sides of a complex potential^9^. Among them, coherent perfect absorption (*CPA*)^10^, which refers to complete absorption of both incident waves, is being extensively investigated^11^,^12^,^13^,^14^,^15^,^16^,^17^,^18^. The interest in *CPA* stems not only for fundamental reasons^10^,^11^,^13^, since it can be interpreted as the time-reversed counterpart of lasing and related to parity-time (*PT*) symmetry^19^, but also in view of its potential applications. Such efforts have spurred investigations and experiments in various areas that span, among others, absorption enhancement^20^, perfect energy feeding into nanoscale systems^21^, intersubband polaritons^22^, slow light waveguides^23^, graphene-based perfect absorbers^24^,^25^,^26^,^27^, and Fano resonant plasmonic metasurfaces^28^.

The concepts of *one-sided reflectionless* and *coherent perfect absorption* have remained so far separated from each other, probably because of the lack of suitable physical systems in which both features would be accessible. Here, we show how a lossy medium that exhibits *ORL* can in general also exhibit *CPA*. The connection is general, not restricted to *PT* symmetric media and could be easily observed in a realistic 1D lossy medium through smooth deformations of the system’s externally tunable parameters. We further argue how this connection, intrinsic to the structure of non-Hermitian degeneracies of scattering matrix *S*, can actually be extended to all points of a *CPA-line*. Such a line is a novel topological structure of non-Hermitian optical media predicted to occur next to a *ORL-line*. Although there has been a number of recent advances in each of these areas of research, particularly restricted to the case of *PT* symmetric media requiring a balance of loss and gain^29^,^30^,^31^,^32^,^33^,^34^, one-sided reflectionless and coherent perfect absorption – *taken together* – may lead to a more complete understanding of non-Hermitian optics in a large class of materials where absorption plays a key role for applications. Photodetectors, photovoltaics and non-reciprocal optical devices just to mention a few instances. The connection we present here is fairly general, hinges on non-Hermitian scattering degeneracies with common notions from quantum mechanics and, though clearly relevant to optics in view of one-way mirrors, cloaks of invisibility and coherent laser absorbers, may well be relevant to unusual phenomena recently observed for acoustic waves^35^,^36^,^37^,^38^,^39^,^40^,^41^.

## ORL and CPA

The scattering properties of a 1D-medium are fully determined by the complex amplitudes *t* = *t*_L_ = *t*_R_, *r*_L_ and *r*_R_ respectively for (reciprocal) transmission and reflection upon incidence from the left (*L*) or from the right (*R*). *ORL* means that *r*_L_ = 0 with *r*_R_ ≠ 0 (or vice versa). The *CPA* condition corresponds, instead, to a specific configuration of input beams, incident at the same time one from the left and one from the right with a definite phase relationship, which are completely absorbed by the sample. Thus, for this configuration of input beams, the output beams to the right and to the left are *both vanishing*. This means that the *CPA* input beams represent an eigenvector of the scattering matrix *S* with eigenvalue zero. As discussed below, the *CPA* condition can finally be stated as *t*^2^ = *r*_R_*r*_L_, *i.e.* det *S* = 0^10^ (see Eq. (3)).

Thus, the main focus of the work is how to connect in general the two conditions *r*_L_ = 0 (*ORL*) and *t*^2^ = *r*_R_*r*_L_ (*CPA*) upon smooth deformations of medium’s external driving parameters. More specifically, for a lossy 1D-photonic crystal, the scattering properties near Bragg reflection can be described^5^,^4^ by the following model susceptibility


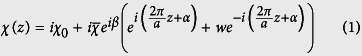


with *χ*_0_, 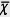, and *w* being non negative real parameters, *a* the crystal period and the phases {*α*, *β*} defined within the interval [0, *π*]. The real part of the spatially independent background susceptibility is ignored for simplicity as it plays no significant role, while its imaginary part *χ*_0_ should be large enough with respect to 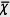 to have everywhere a lossy medium, *i.e.*, 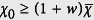. In this rather generic model, the *ORL* condition (*r*_L_ = 0) is simply attained when *w* = 0^5^,^4^, in which case the real and imaginary parts of the susceptibility modulation *χ*(*z*) − *iχ*_0_ are spatially shifted by *π*/2 and satisfy the spatial Kramers-Kronig relations^6^. The reflection and transmission of a light beam with a wave-vector 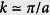 can be described on the basis of a minimal coupled-mode model accounting for Bragg scattering in a sample of length *L* ≫ *a*, as usual. Then, the *CPA* condition is attained when


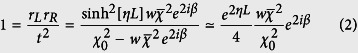


where 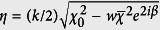 (with *Re*[*η*] > 0 due to losses). The last term in Eq. (2) holds when |*e*^*ηL*^| ≫ 1 (|*t*| ≪ 1) and *w* ≪ 1, and this is precisely the regime we are interested in as it can occur near a *ORL* point in a lossy medium. It thus appears that, while the parameter *α* is immaterial, the *CPA* condition can in general be satisfied only if *β* can be tuned at will within the whole interval [0, *π*], regardless of the value of *w*. In fact, although 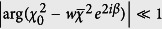, 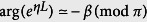 need not be small at the *CPA* point as *kL* ≫ 1.

**Figure 1:**
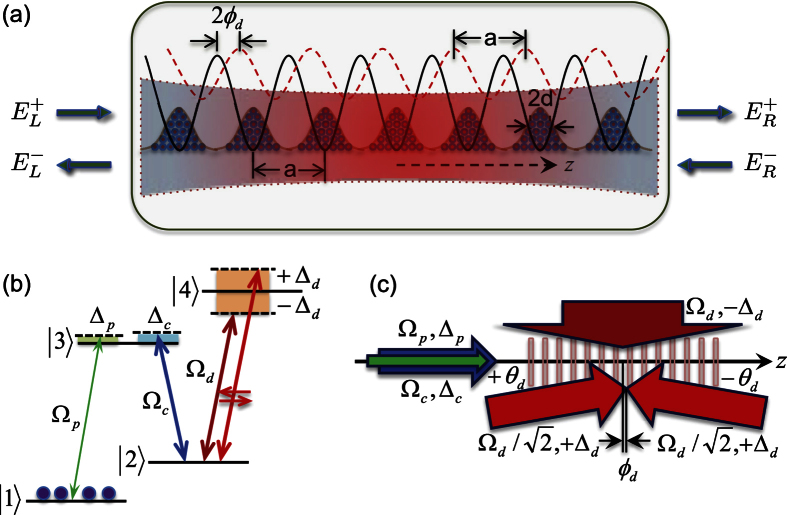
*The CPA* – *ORL connection* scattering scheme. (**a**) Cold ^87^Rb atoms are loaded in a *1D* optical lattice (*black-solid*) of period a. These atoms suffer a dynamic level shift (*red-dashed*) with the same periodicity, but phase shifted with respect to the optical lattice. The incident *probe* electric field amplitudes (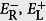) are scattered by the atomic lattice into the outgoing electric field amplitudes (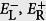). For fields (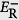) incident from the right, *e.g.*, outgoing amplitudes consist of waves (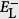) transmitted with amplitude *t*_R_ in the −*z* direction as well as waves (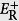) reflected with amplitude *r*_R_ in the +*z* direction; likewise for fields (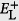) incident from the left and reflected (transmitted) with amplitude *r*_L_ (*t*_L_); while in general *r*_L_ ≠ *r*_R_, *t*_L_ = *t*_R_ = *t*. (**b**) A four-level *N*-configuration through which ^87^Rb atoms are driven by a *weak* near-resonant probe field (*green*) on the 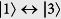 transition, a *moderate* resonant coupling field (*blue*) on the 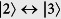 transition and a *strong* far-detuned dressing field (*red*) on the 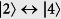 transition. (**c**) The probe, with Rabi frequency Ω_*p*_ and detuning Δ_*p*_, and the resonant coupling (Δ_*c*_ = 0), with Rabi frequency Ω_*c*_, propagate in the *z* direction. The dressing field has instead a TW component propagating in the *x* direction, with Rabi frequency Ω_*d*_ and detuning −Δ_*d*_, and a SW component modulated in the *z* direction, with detuning +Δ_*d*_.

Though solid-state photonic structures may be considered^4^, coherently-prepared multi-level atoms^5^,^42^ are attractive for exploring non-Hermitian optics, because of the easy reconfiguration of the scattering process through well established control techniques enabled by electromagnetically induced transparency (EIT)^43^. In fact, the realization of atomic platforms to investigate non-Hermitian models is currently a very active experimental endeavor^44^,^45^. We consider the realistic atomic system of Fig. 1, which provides an implementation of the model of Eq.(1). The photonic crystal consists of cold atoms coherently driven by a near-resonant probe beam (Ω_*p*_, Δ_*p*_ ≈ 0), a resonant coupling beam (Ω_*c*_, Δ_*c*_ = 0) and an far-detuned dressing field (Ω_*d*_,|Δ_*d*_| ≫ 0). The latter has both a traveling-wave (TW) and a standing-wave (SW) components with opposite detunings and induces on level |2〉 a dynamic shift 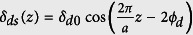, where 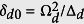 and the phase shift 2*ϕ*_*d*_ is relative to the optical lattice modulating the atomic density. As a matter of fact, by adjusting only three of the above independent control parameters, namely {Δ_*p*_, *δ*_*d*0_, *ϕ*_*d*_}, it is possible to identify scattering processes for which the existence of the *CPA*–*ORL* connection can be proven. More specifically, this is done by solving the density matrix equations for the atomic level configuration of Fig. 1 whose matrix elements will depend, among other parameters kept fixed here as in Fig. 6 of ref. 45, on the three parameters (Δ_*p*_*, δ*_*d*0_*, ϕ*_*d*_) (See sect. II of ref. 45). For each choice of these three experimentally tunable parameters, we numerically compute the full susceptibility χ(*z*), which can be cast in the form of Eq. (1) when its higher order Fourier components are disregarded. From χ(*z*) we then directly obtain through transfer matrix calculations^46^ the scattering amplitudes t, r_L_ and r_R_ that identify a specific scattering process.

**Figure 2:**
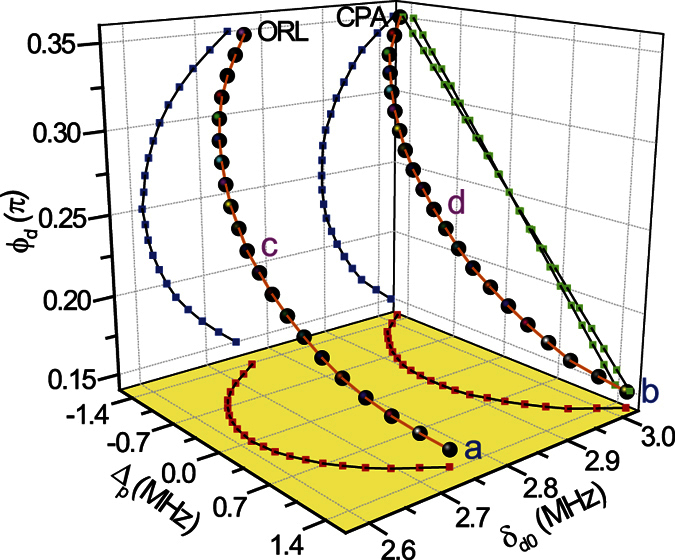
A *CPA*-line and the nearby *ORL*-line for a typical photonic crystal structure are shown in the parameter space {Δ_*p*_, *δ*_*d*0_, *ϕ*_*d*_}. The two lines which are nearly “parallel” are also shown projected onto the {*δ*_*d*0_, *ϕ*_*d*_} plane (*blue lines*), the {Δ_*p*_, *ϕ*_*d*_} plane (*green lines*) and the {Δ_*p*_, *δ*_*d*0_} plane (*red lines*). The points labeled (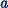), (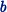), (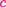) and (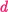) correspond to those marked in Fig. 3.

**Figure 3:**
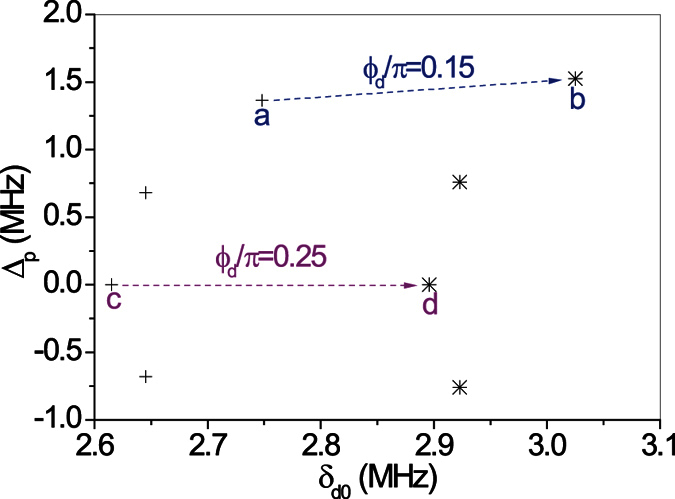
Pairs of *ORL* (+) and CPA (*) points in the {Δ _*p*_, *δ*_*d*0_} plane (yellow plane in Fig. 2) corresponding to values of *ϕ*_*d*_ ranging from 0.15 × *π* to 0.30 × *π* (top to bottom).

**Figure 4:**
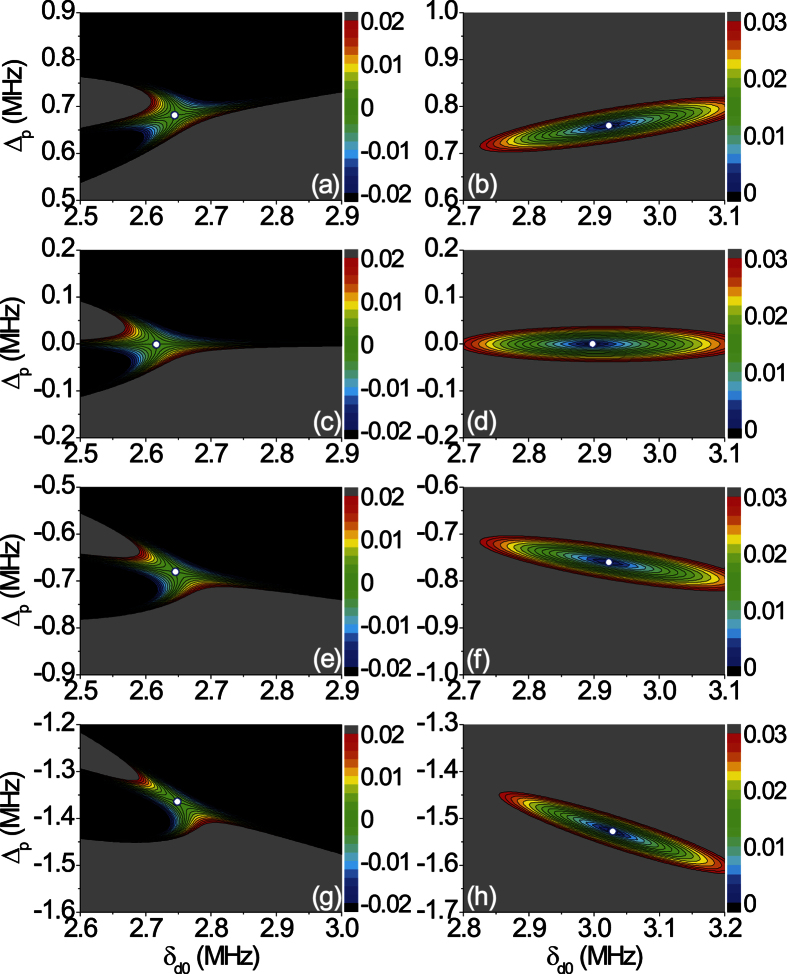
**a–c,e–g** (left column)] Contour plots of *Re*[*r*_*L*_] × *Im*[*r*_*L*_]: *ORL*-points (*white-dots*) occur when both *Re*[*r*_*L*_] and *Im*[*r*_*L*_] change sign in the {Δ_*p*_, *δ*_*d*0_}-plane. [**b**–**d**,**f**–**h** (right column)] Corresponding *CPA*-points (*white-dots*) occur when |*t*^2^ − *r*_*L*_*r*_*R*_| vanishes. Each pair of *ORL*-*CPA* points is found for a given value of *ϕ*_*d*_ (from top to bottom: *ϕ*_*d*_/*π* = 0.20, 0.25, 0.30, 0.35).

A relevant sets of *ORL* points (*r*_L_ = 0) and the associated *CPA*-points (*t*^2^ = *r*_R_*r*_L_) are reported in the 3D parameter space {Δ_*p*_, *δ*_*d*0_, *ϕ*_*d*_} of Fig. 2. A CPA-line lying roughly parallel to an ORL-line is shown there. Hence, we can access a CPA-point starting from a ORL-point essentially by adjusting the parameter δ_*d*0_. The reason is simply that (i) the transmission amplitude t is always small in our lossy atomic medium and (ii) the reflection amplitudes r_*L*_ and r_*R*_ are more sensitive to *δ*_*d*0_ than Δ_*p*_ at a fixed value of *ϕ*_*d*_. A range of *ϕ*_*d*_ values centered at *ϕ*_*d*_ = *π*/4 is shown, being our system periodic in *ϕ*_*d*_ with period *π*, while varying *ϕ*_*d*_ from *ϕ*_*d*_ = *π*/4 to 3*π*/4 (or to −*π*/4) simply changes the reflectionless behavior from the “left” into reflectionless from the “right”. Notice also that the *CPA*-lines and *ORL*-lines are symmetric under the simultaneous changes *ϕ*_*d*_ → *π*/2−*ϕ*_*d*_ and Δ_*p*_ → −Δ_*p*_. We can *always* find an isolated *CPA*-point associated to a nearby isolated *ORL*-point through cuts along {Δ_*p*_, *δ*_*d*0_}-planes as shown in Fig. 3. Figure 4 illustrates further examples of how *ORL*-points and the associated *CPA*-points are computed. *ORL*-points are characterized by *r*_*L*_ = 0 and are here obtained by solving the two real equations *Re*[*r*_*L*_] = 0 and *Im*[*r*_*L*_] = 0. In the neighborhood of a solution both *Re*[*r*_*L*_] and *Im*[*r*_*L*_] change sign and their product changes sign in four alternating sections (i.e., deformed quadrants) of the {Δ_*p*_, *δ*_*d*0_}-plane as shown in Fig. 4(a–c,e–g). This corresponds to the fact that the phase of *r*_*L*_ varies by 2*π* when a *ORL*-point is encircled in the {Δ_*p*_, *δ*_*d*0_}-plane, which embodies the freedom of choice of *β* in Eq. (2), and is a key point as discussed below. *CPA*-points, characterized by *t*^2^ = *r*_*L*_*r*_*R*_, are illustrated instead in Fig. 4(b–d,f–h) as minima of the function |*t*^2^ − *r*_*L*_*r*_*R*_|.

## Discussion

The *CPA* – *ORL* connection can also be assessed in more general terms starting from the two-ports scattering process,


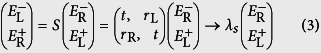


where the *S* matrix relates the outgoing (electric) field amplitudes 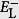 and 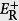 to the incoming (electric) field amplitudes 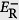 and 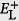 (see Fig. 1a). The eigenvalues and eigenvectors of *S* are obtained through the last term in Eq. (3). It is here worth noting that we have chosen one of the most common representations of the *S* matrix, the other one having instead *r*_L_ and *r*_R_ on the diagonal. While the scattering is solely determined by the measurable complex amplitudes *t*, *r*_L_ and *r*_R_ and all physical results are independent of which *S* matrix representation is used, the specific choice of *S* in Eq. (3) is appropriate to prove the *CPA* – *ORL* connection, where the *ORL* condition is in this case directly related to a non-Hermitian degeneracy (or exceptional point) of *S*, as we illustrate in the following.

**Figure 5:**
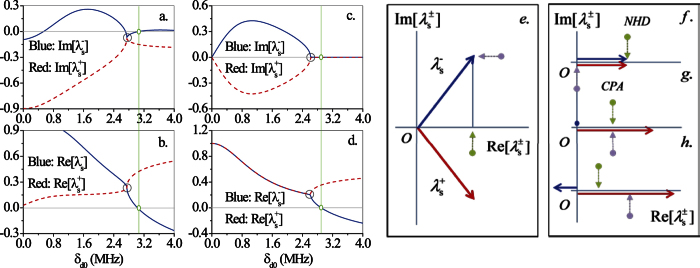
*Coherent Perfect Absorption (CPA*) and *Non-Hermitian Degeneracies (NHD)*. Typical topology of the *S*-matrix eigenvalues (4) around a *NHD* (circle) for non-Hermitian (**a**,**b**) and pseudo-Hermitian (**c**,**d**) scattering processes in the photonic crystal structure of Fig. 1. Vertical green line indicate *CPA*-points *next* to a *NHD*-point respectively at (**a**,**b**) *δ*_*d*0_ = 3.02 MHz (with Δ_*p*_ = 1.52 MHz, point (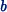) in Fig. 3) and *δ*_*d*0_ = 2.75 MHz (with Δ_*p*_ = 1.36 MHz, point (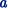) in Fig. 3) and at (**c**,**d**) *δ*_*d*0_ = 2.89 MHz (with Δ_*p*_ = 0, point (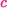) in Fig. 3) and *δ*_*d*0_ = 2.61 MHz (with Δ_*p*_ = 0, point (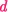) in Fig. 3). Polar representation of the two eigenvalues before (**e**) and at (**f** ) the *NHD*-point, and at (**g**) and after (**h**) the *CPA*-point for the case (**c**,**d**) (with Δ_*p*_ = 0). Light green and violet arrows mark respectively the two eigenvalues half-sum (*t*) and half-difference (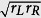).

In general, *S* is non-Hermitian, its eigenvalues


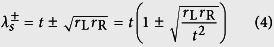


are complex and the (unnormalized) eigenvectors 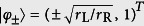 are not orthogonal. *Non-Hermitian degeneracies* of *S* occur when the eigenvalues merge into one another [Fig. 5(a–d)] and the eigenvectors coalesce into a single state^7^, being the *S* matrix no longer diagonalizable. The two coalescing eigenvalues are analytically connected by a square-root branch-point, with associated Riemann sheets, and are physically associated with unidirectional reflectionless scattering states occurring when *r*_L_ = 0 (or *r*_R_ = 0)^4^,^5^. For a non-Hermitian matrix, degeneracies are of codimension two, that is points in a two-parameter space (*NHD*-point) and curves in a three-parameter space (*NHD*-line). Meanwhile, *CPA* occurs when either one of the two eigenvalues 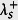 or 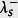 vanishes [Fig. 5(a–d)] along with the determinant of *S* (this condition is independent of the specific choice of *S* matrix representation). The corresponding eigenvector describes a perfect absorption state^10^ with amplitudes and phases of the incoming fields from the left and from the right precisely chosen so that no outgoing light intensity can be observed^13^,^18^.

We start providing an intuitive illustration of how *CPA* and *ORL* are connected with one another in the particular, but important, case for which (*i*) the reflection phases are such that *ϕ*_*L*_ + *ϕ*_*R*_ = {0, *π*} and (*ii*) the transmission amplitude *t* is real. The corresponding eigenvalues are either real or complex conjugate in pairs depending on whether the two phases add up to 0 or to *π* [Fig. 5(c,d)]. Thus (half) sum of the two eigenvalues represents *t* and can be depicted, as we move in the parameter space toward degeneracy, by a vector whose magnitude decreases along the real axis of Fig. 5(e) for decreasing transmission. So does (half) difference of the two eigenvalues representing the geometric mean of *r*_*L*_ and *r*_*R*_, which can be depicted by a vector parallel to the imaginary axis. As we move through degeneracy, the eigenvalues sum will keep decreasing *but* their difference will increase after moving away from zero (degeneracy) [Fig. 5(f)] owing to the intrinsic bifurcation (topological) structure of the branch-point. Hence there will *always* be a point where sum and difference will be equal (to each other), *i.e.*, 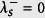 [Fig. 5(g)]. It is worth noting that under the conditions (*i*.) and (*ii*.) an Hermitian invertible transformation *η* exists indeed for which the adjoint of the (non-Hermitian) scattering matrix *S* satisfies *S*^†^ = *ηSη*^−1^, *i.e.*, *S* is *pseudo-Hermitian*^48^. The reverse is also true and hence the pseudo-Hermiticity of *S* is the basic mathematical structure responsible for the direct connection between the *ORL* and the *CPA* point, *at least* for the specific spectrum of *S* shown in Fig. 5(c,d). Note that this particular case – realized in the all-optically tunable atomic system of Fig. 1 simply setting Δ_*p*_ = 0 – is essentially analogous to a *PT* symmetric one, even though our system is *always* lossy, both before and after the *NHD* point.

Yet, a *CPA*-point can be typically found in the vicinity of a *ORL*-point under more general conditions and, in particular, without restricting ourselves to pseudo-Hermiticity. For definiteness we take the *NHD*-point at 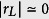 assuming, without loss of generality, that around this point |*r*_*R*_| and |*t*| are nonvanishing. For lossy media we may further take |*t*| ≪ 1, with |*r*_*R*_| being in general on the order of unity^5^. The perfect absorption condition 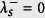 is satisfied when *r*_*L*_*r*_*R*_ = *t*^2^, *i.e.* when


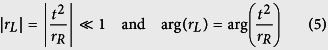


are both satisfied, implying that |*r*_*L*_| and arg(*r*_*L*_) should be *independently* adjusted (just as the phase *β* in Eq. (2) should be tuned at will, regardless of the value of *w*). Note that the CPA conditions in Eq. (5) generalize those given above for the pseudo-Hermitian case, and are only restricted by the requirement that |*r*_*L*_| be small at the *CPA*-point, which occurs when this point is associated to a nearby *ORL*-point. In general, we do expect *t*^2^/*r*_*R*_ to be smoothly varying in the vicinity of this point while arg(*r*_*L*_) can be varied at will when the parameters defining the system are smoothly changed so to *encircle* the *ORL*-point, *i.e.* the *NHD* of *S*^5^. A simple geometric illustration of this property similar to that provided in Fig. 5(e–h) is not so viable in the general, non pseudo-Hermitian case (such as that of Fig. 5(a,b)); yet, a direct analytical argument shows that |*r*_*L*_| and arg(*r*_*L*_) can be independently adjusted when encircling the *ORL*-point.

In a typical scattering process, *r*_*L*_ depends smoothly on several experimental parameters. We consider here how the real (*u*) and the imaginary (*v*) parts of *r*_*L*_ vary near the *ORL*-point as a function of only two of these parameters, keeping all other ones fixed. In terms of these two parameters, say *x* and *y*, one has


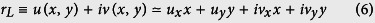


where the partial derivatives *u*_*x*_ = ∂*u*/∂*x*, *u*_*y*_ = ∂*u*/∂*y*, *v*_*x*_ = ∂*v*/∂*x*, and *v*_*y*_ = ∂*v*/∂*y* are evaluated at the *ORL*-point taken at (*x*, *y*) = (0, 0). Note that it is not needed to combine *x* and *y* into a single complex parameter *x* + *iy* as *r*_*L*_ is not assumed to be holomorphic here. When *u*_*x*_*v*_*y*_ − *v*_*x*_*u*_*y*_ ≠ 0, it is always possible to select *x* and *y* to obtain any required values of arg(*r*_*L*_) and of |*r*_*L*_|, *provided* the latter is small enough that higher order terms in Eq. (6) are indeed negligible. Thus, under typical circumstances we expect a *CPA* and a *ORL* points to be close to each other in a scattering process from lossy media with |*t*| small. For example, in Fig. 3 the case *ϕ*_*d*_ = 0.25 × *π (pink-arrow*) represents changes in the scattering matrix as one moves from its *NHD*-point (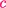) to its *CPA*-companion (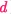), namely for a pseudo-Hermitian matrix (Δ_*p*_ = 0). Similarly, the case *ϕ*_*d*_ = 0.15 × *π (blue-arrow*) represents changes as one moves from the *NHD*-point (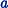) to its *CPA*-companion (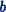), namely for the general non-Hermitian case. Actually, the case in which *u*_*x*_*v*_*y*_ − *v*_*x*_*u*_*y*_ = 0 cannot be excluded. Assuming that (*u*_*y*_, *v*_*y*_) ≠ (0, 0) and writing (*u*_*x*_, *v*_*x*_) = *μ*(*u*_*y*_, *v*_*y*_) with *μ* real, one then has


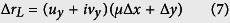


which implies that, while |*r*_*L*_| = |Δ*r*_*L*_| can be varied, arg(*r*_*L*_) = arg(Δ*r*_*L*_) is fixed because 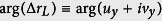. In this case, we expect to find no *CPA*-point in the vicinity of a *ORL*-point when all other parameters are kept constant. Clearly, also when higher order terms in the above expansion of *r*_*L*_ become important as for instance in the peculiar case where all partial derivatives in Eq. (6) are vanishingly small, the occurrence of the *CPA* point is not granted.

Defining *ρe*^*iθ*^ ≡ −*r*_*L*_/*t*, the scattering matrix eigenvector at the *CPA*-point, where the corresponding eigenvalue 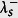 vanishes, can be eventually written as,


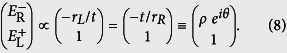


**Figure 6:**
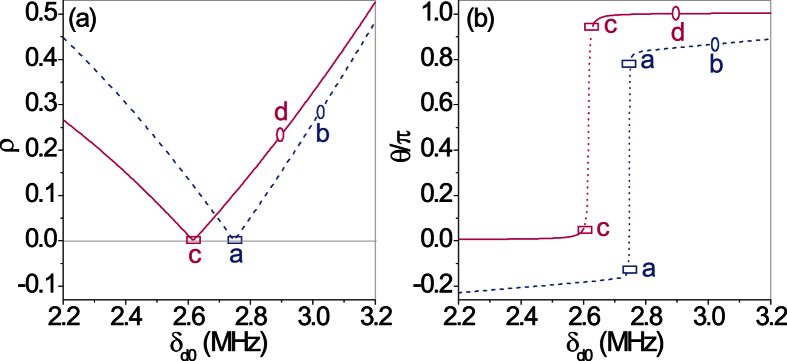
Plots of *ρ* (**a**) and *θ* (**b**) along the directions marked by the two color-dashed arrows in Fig. 3(a) for {*ϕ*_*d*_ = 0.15 × *π*, Δ_*p*_ = 0.576*δ*_*d*0_ − 0.220 MHz} (*blue-dashed* line) and for {*ϕ*_*d*_ = 0.25 × *π*, Δ_*p*_ = 0.0 MHz} (*red-solid* line). The two *CPA*-points (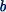) and (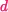) (circles) placed at *δ*_*d*0_ = 3.025 MHz (*ϕ*_*d*_ = 0.15 × *π*) and at *δ*_*d*0_ = 2.896 MHz (*ϕ*_*d*_ = 0.25 × *π*) correspond to those shown respectively in Fig. 5(a,b) (non-Hermitian) and Fig. 5(c,d) (pseudo-Hermitian). The two *ORL*-points (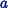) and (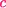) (squares) placed at *δ*_*d*0_ = 2.748 MHz (*ϕ*_*d*_ = 0.15 × *π*) and at *δ*_*d*0_ = 2.615 MHz (*ϕ*_*d*_ = 0.25 × *π*) correspond to those shown respectively in Fig. 5(a,b) (non-Hermitian) and Fig. 5(c,d) (pseudo-Hermitian). At the *ORL*-points the phase *θ* is not defined and changes by *π*, as shown by vertical dotted lines in panel (b). At the *CPA*-point (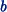) the ratio of right to left incoming intensities is about 0.082 while at the *CPA*-point (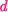) the ratio is 0.053.

The complex quantity *ρe*^*iθ*^ is examined in Fig. 6 both for the pseudo-Hermitian and non-Hermitian cases. At the *CPA*-point, the eigenvector’s components scale as 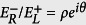, with *ρ* ≪ 1 according to Eq. (5). Both modulus (*ρ*) and phase (*θ*) of the (small) incoming field from the right, with respect to the incoming field from the left (i.e, the nearly reflectionless side), should be properly chosen to observe the typical perfect absorption behavior. Since the *CPA*-point considered here is associated to a *ORL* point, in general, perfect absorption requires very unbalanced incoming fields. As a matter of fact, the characteristic destructive interference conditions leading to perfect absorption for light scattering in both directions occur here for very unbalanced right and left reflectivities |*r*_*R*_| ≫ |*r*_*L*_|. In turn, a tiny input field from the right is sufficient to ensure that the outgoing field to the left vanishes, while a large input field from the left is necessary to destructively interfere with the reflected field from the right side. This *CPA* configuration provides, in particular, a high-contrast reflectivity control of a test beam incident from the right via a pump beam incident from the left.

## Conclusions

A new insight into the non-Hermitian optics of a familiar class of lossy photonic crystals is here discussed. Through continuous deformations of the scattering matrix *S* around a one-sided reflectionless (*ORL*) point, a *CPA* point can be typically attained. Nearby pairs of *ORL* and *CPA* “points” or even “lines” appear, respectively, through controlling the crystal 2D or 3D parameter space. In such cases, the *CPA* scattering states associated to *ORL* points turn out to be significantly unbalanced, indicating a dynamically reversible high-contrast reflectivity control of the input beams. Finally, while the results here presented refer to realistic atomic structures^44^,^45^, our general discussion can be easily adapted to atomic-like multilevel centers^49^ in solids, such as NV diamond or rare-earth-doped crystals, also allowing for EIT control of light scattering^50^,^51^. Hence the optics of photonic crystals is poised to have a privileged place in assessing that not only standard Hermitian models but also a broad set of non-Hermitian ones are bound to have physical interpretations.

## Additional Information

**How to cite this article**: Wu, J.-H. *et al.* Coherent perfect absorption in one-sided reflectionless media. *Sci. Rep.* **6**, 35356; doi: 10.1038/srep35356 (2016).
